# Draft genome sequence of the endophytic fungus *Phoma* sp. strain YAFEF320, isolated from *Gerbera jamesonii*

**DOI:** 10.1128/mra.00907-23

**Published:** 2023-12-05

**Authors:** Zhenhong Lu, Xiaolong Yuan, Chuanguang Zhang, Shenchong Li, Chunmei Yang, Yi Wang

**Affiliations:** 1 Flower Research Institute, Yunnan Academy of Agricultural Sciences, Kunming, Yunnan, China; 2 Laboratory of Forest Plant Cultivation and Utilization, Yunnan Academy of Forestry and Grassland, Kunming, Yunnan, China; University of California, Riverside, Riverside, California, USA

**Keywords:** *Phoma *sp., genome, *Gerbera jamesonii*

## Abstract

Here, we report a draft genome sequence of endophytic fungus *Phoma* sp*.* strain YAFEF320, isolated from the roots of *Gerbera jamesonii*. The genome size of *Phoma* sp. YAFEF320 was 32,542,820 bp with 52.08% GC content. The genome resource will support future research into potential secondary metabolite diversity of this fungus.

## ANNOUNCEMENT

Endophytic species of the fungus *Phoma* can produce abundant bioactive metabolites, including polyketides, terpenes and terpenoids, thio-diketopiperazines, macrosporin, isocoumarin and cytochalasin derivatives, phenolic compounds, and alkaloids (1 – 3). These bioactive metabolites have anti-proliferative (4), antiviral (5), antimicrobial (6) anti-inflammatory (7, 8) anticancer (9, 10), and phytotoxic activities (11, 12). Several reviews have discussed the potential values for applying these species, including their bioherbicidal (13), antimicrobial (14), and anticancer properties (10). Three genomes of *Phoma* spp. were sequenced, including those of *Phoma herbarum* JCM15942 and *Phoma* sp. strains XZ068 and RAV-16-625 (15). BII-Rafflesfungin and a humulene biosynthetic gene cluster were identified by genome mining (16). In this study, we report a new genome of *Phoma* sp. YAFEF320 from the roots of Barberton daisy (*Gerbera jamesonii*).

*Phoma* sp. strain YAFEF320 was isolated from the roots of *G. jamesonii*. The isolation process was as follows. The roots were collected from the Gerbera planting base, Yunnan Academy of Agricultural Sciences, Yunnan Province, China (25°18′11″ N, 102°85′14″ E) in 2020 and soaked in tap water for 24 h to clean them. They were then rinsed with sterile water for 5 min. The roots were ground in a ball mill (GT100; Grinder, Beijing, China) at 180 rpm and 25°C, and sterile water was added to obtain a slurry. Finally, the root slurry was diluted and spread onto solid potato dextrose agar (PDA) with 100 µg/mL ampicillin, kanamycin, and streptomycin. Single colonies were transferred to fresh media after 5 days of incubation at 28°C. A single colony (strain YAFEF048) was identified by sequencing its amplified ITS region with ITS1 and ITS2 primers and comparing it with the known ITS region of *Phoma* to identify the new *Phoma* sp. The ITS sequence was deposited in GenBank (accession number: OR487462), and *Phoma* sp. strain YAFEF320 was deposited in the China Center for Type Culture Collection (deposit number: CCTCC M 20211093).

The mycelia of this strain were harvested from filter paper after 5 days of cultivation at 28°C in liquid PDA on a rotary shaker at 150 rpm. Its genomic DNA was extracted using a DNeasy Mini Kit (Qiagen, Valencia, CA, USA) following the manufacturer’s instructions. Illumina (San Diego, CA, USA) sequencing libraries were prepared using a TruSeq DNA Sample Prep Kit (Illumina) with insert sizes of 400 bp. The library was sequenced on an Illumina NovaSeq 6000 platform with a 2 × 150 bp paired-end configuration. A total of 48,949,704 raw reads were obtained (Table 1). High-quality cleaned sequences (7.22 Gb; coverage, 225×) were obtained by trimming the adapter sequences with AdapterRemoval version 2 (17). The *de novo* genome assembly was conducted using SPAdes version 3.10.1 (18). The default parameters were used for all the software unless otherwise specified. The genome assembly size of the YAFEF320 isolate was 32,542,820 bp with 76 scaffolds. The longest scaffold was 2,364,249 bp, with an N50 value of 1,076,372 bp and an N90 value of 326,747 bp. The GC content was 52.08%. The completeness of the genome assembly was assessed using BUSCO version 5.4.2 (19), and its completeness value was 97.2%.

**TABLE 1:** Genome sequencing statistics

Parameter	Value
Raw reads	
Read number	48,949,704
Coverage	225×
Genome assembly	
Total base	32.5 Mb
Total scaffolds	76
Max sequence length	2,364,249 bp
N50	1,076,372 bp
N90	326,747 bp
Genome assembly assessment	
Complete BUSCOs	97.20%
Complete and single-copy BUSCOs	96.90%
Complete and duplicated BUSCOs	0.30%

## Data Availability

This study project is available under NCBI BioProject accession number PRJNA1011515 and BioSample accession number SAMN37222078. The complete genome assembly of *Phoma* sp. is available under GenBank accession number JAVLWG000000000. The Illumina NovaSeq sequencing raw data were deposited in GenBank under the accession number SRX21629953.

## References

[1] Kong F , Wang Y , Liu P , Dong T , Zhu W . 2014. Thiodiketopiperazines from the marine-derived fungus Phoma sp. OUCMDZ-1847. J Nat Prod 77:132–137. doi:10.1021/np400802d 24370114

[2] Chen Y , Yang W , Zou G , Chen S , Pang J , She Z . 2019. Bioactive polyketides from the mangrove endophytic fungi Phoma sp. SYSU-SK-7. Fitoterapia 139:104369. doi:10.1016/j.fitote.2019.104369 31626911

[3] Rai M , Gade A , Zimowska B , Ingle AP , Ingle P . 2018. Marine-derived Phoma-the gold mine of bioactive compounds. Appl Microbiol Biotechnol 102:9053–9066. doi:10.1007/s00253-018-9329-2 30187101

[4] Ku H , Baek J-Y , Kang KS , Shim SH . 2022. A new anti-proliferative compound from an endophytic fungus, Phoma sp. Nat Prod Res 36:5584–5590. doi:10.1080/14786419.2021.2022663 34965790

[5] Liu S-S , Jiang J-X , Huang R , Wang Y-T , Jiang B-G , Zheng K-X , Wu S-H . 2019. A new antiviral 14-nordrimane sesquiterpenoid from an endophytic fungus Phoma sp.. Phytochemistry Letters 29:75–78. doi:10.1016/j.phytol.2018.11.005

[6] Zhang H , Xiong Y , Zhao H , Yi Y , Zhang C , Yu C , Xu C . 2013. An antimicrobial compound from the endophytic fungus Phoma sp. isolated from the medicinal plant Taraxacum mongolicum. J Taiwan Inst Chem Eng 44:177–181. doi:10.1016/j.jtice.2012.11.013

[7] Lee M-S , Wang S-W , Wang G-J , Pang K-L , Lee C-K , Kuo Y-H , Cha H-J , Lin R-K , Lee T-H . 2016. Angiogenesis inhibitors and anti-inflammatory agents from Phoma sp. NTOU4195. J Nat Prod 79:2983–2990. doi:10.1021/acs.jnatprod.6b00407 27976895

[8] Kim JW , Ko W , Kim E , Kim GS , Hwang GJ , Son S , Jeong M-H , Hur J-S , Oh H , Ko S-K , Jang J-H , Ahn JS . 2018. Anti-inflammatory phomalichenones from an endolichenic fungus Phoma sp.. J Antibiot 71:753–756. doi:10.1038/s41429-018-0058-7 29700423

[9] Yu C , Nian Y , Chen H , Liang S , Sun M , Pei Y , Wang H . 2022. Pyranone derivatives with antitumor activities, from the endophytic fungus Phoma sp. YN02-P-3. Front Chem 10:950726. doi:10.3389/fchem.2022.950726 35873041 PMC9300907

[10] Rai M , Zimowska B , Gade A , Ingle P . 2023. Phoma spp. an untapped treasure of cytotoxic compounds: current status and perspectives. Appl Microbiol Biotechnol 107:4991–5001. doi:10.1007/s00253-023-12635-9 37401998

[11] Rivero-Cruz JF , Macías M , Cerda-García-Rojas CM , Mata R . 2003. A new phytotoxic nonenolide from Phoma herbarum. J Nat Prod 66:511–514. doi:10.1021/np020501t 12713403

[12] Kalam S , Khan NA , Singh J . 2014. A novel phytotoxic phenolic compound from Phoma herbarum FGCC#54 with Herbicidal potential. Chem Nat Compd 50:644–647. doi:10.1007/s10600-014-1043-4

[13] Rai M , Zimowska B , Shinde S , Tres MV . 2021. Bioherbicidal potential of different species of Phoma: opportunities and challenges. Appl Microbiol Biotechnol 105:3009–3018. doi:10.1007/s00253-021-11234-w 33770245

[14] Rai M , Zimowska B , Gade A , Ingle P . 2022. Promising antimicrobials from Phoma spp.: progress and prospects. AMB Express 12:60. doi:10.1186/s13568-022-01404-y 35604500 PMC9125353

[15] Zhai Y , Li Y , Zhang J , Zhang Y , Ren F , Zhang X , Liu G , Liu X , Che Y . 2019. Identification of the gene cluster for bistropolone-humulene meroterpenoid biosynthesis in Phoma sp.. Fungal Genet Biol 129:7–15. doi:10.1016/j.fgb.2019.04.004 30980906

[16] Sinha S , Nge C-E , Leong CY , Ng V , Crasta S , Alfatah M , Goh F , Low K-N , Zhang H , Arumugam P , Lezhava A , Chen SL , Kanagasundaram Y , Ng SB , Eisenhaber F , Eisenhaber B . 2019. Genomics-driven discovery of a biosynthetic gene cluster required for the synthesis of BII-Rafflesfungin from the fungus Phoma sp. F3723. BMC Genomics 20:374. doi:10.1186/s12864-019-5762-6 31088369 PMC6518819

[17] Schubert M , Lindgreen S , Orlando L . 2016. Adapterremoval V2: rapid adapter trimming, identification, and read merging. BMC Res Notes 9:88. doi:10.1186/s13104-016-1900-2 26868221 PMC4751634

[18] Prjibelski A , Antipov D , Meleshko D , Lapidus A , Korobeynikov A . 2020. Using spades de novo assembler. Curr Protoc Bioinformatics 70:e102. doi:10.1038/s41598-020-75855-3 32559359

[19] Simão FA , Waterhouse RM , Ioannidis P , Kriventseva EV , Zdobnov EM . 2015. BUSCO: assessing genome assembly and annotation completeness with single-copy Orthologs. Bioinformatics 31:3210–3212. doi:10.1093/bioinformatics/btv351 26059717

